# Preparation and Optimization of PEGylated Nano Graphene Oxide-Based Delivery System for Drugs with Different Molecular Structures Using Design of Experiment (DoE)

**DOI:** 10.3390/molecules26051457

**Published:** 2021-03-07

**Authors:** Mohammad Hossain Shariare, Abdullah-Al Masum, Sultan Alshehri, Fars K. Alanazi, Jamal Uddin, Mohsin Kazi

**Affiliations:** 1 Department of Pharmaceutical Sciences, North South University, Dhaka 1229, Bangladesh; mohammad.shariare@northsouth.edu (M.H.S.); abdullah.masum01@northsouth.edu (A.-A.M.); 2 Department of Pharmaceutics, College of Pharmacy, King Saud University, Riyadh 11451, Saudi Arabia; salshehri1@ksu.edu.sa (S.A.); afars@ksu.edu.sa (F.K.A.); 3 Center for Nanotechnology, Department of Natural Sciences, Coppin State University, Baltimore, MD 21216, USA; juddin@coppin.edu

**Keywords:** graphene oxide (GO), nano graphene oxide (NGO), NGO-PEG, aromatic ring, loading efficiency, methotrexate, diclofenac, acetaminophen

## Abstract

Graphene oxide (GO), due to its 2D planar structure and favorable physical and chemical properties, has been used in different fields including drug delivery. This study aimed to investigate the impact of different process parameters on the average size of drug-loaded PEGylated nano graphene oxide (NGO-PEG) particles using design of experiment (DoE) and the loading of drugs with different molecular structures on an NGO-PEG-based delivery system. GO was prepared from graphite, processed using a sonication method, and functionalized using PEG 6000. Acetaminophen (AMP), diclofenac (DIC), and methotrexate (MTX) were loaded onto NGO-PEG particles. Drug-loaded NGO-PEG was then characterized using dynamic light scattering (DLS), Fourier transform infrared (FTIR), scanning electron microscopy (SEM), differential scanning calorimetry (DSC), XRD. The DLS data showed that the drug-loaded NGO-PEG suspensions were in the size range of 200 nm–1.3 µm. The sonication time and the stirring rate were found to be the major process parameters which affected the average size of the drug-loaded NGO-PEG. FTIR, DSC, XRD, and SEM demonstrated that the functionalization or coating of the NGO occurred through physical interaction using PEG 6000. Methotrexate (MTX), with the highest number of aromatic rings, showed the highest loading efficiency of 95.6% compared to drugs with fewer aromatic rings (diclofenac (DIC) 70.5% and acetaminophen (AMP) 65.5%). This study suggests that GO-based nano delivery systems can be used to deliver drugs with multiple aromatic rings with a low water solubility and targeted delivery (e.g., cancer).

## 1. Introduction

The development of nanosystems has attracted much research attention and enjoyed tremendous growth worldwide. Several different types of nanosystems, such as silica nanoparticles [1], liposomes [2], and metal-based and carbon-based nanomaterials [3,4], have been developed since the emergence of nanosystems. Carbon-based nanosystems, carbon nanotubes (CNTs) [5] and graphene or its oxidation derivatives, graphene oxide (GO) [6], are sought after for their physical and chemical properties and have been extensively studied [7,8]. CNTs and GO have some similar behaviors [9] but, in comparison, GO may provide some advantages over CNTs. For example, the toxicity of GO (in vitro) in U251 human glioma cells is lower than that of CNTs [10,11]. The 2D plane structure of GO allows for the loading of microspheres with a diameter larger than several hundred nanometers [12]. The larger surface area of GO improves the interfacial contact and prevents aggregation [13,14]. The formation of stable colloidal dispersions in the solvent allows GO to be processed easily [15]. The surface chemistry (functional groups) of GO suggests its polar characteristics, and GO is highly stable owing to the strong covalent bond between its carbon atoms [16,17]. Due to GO’s favorable physical and chemical properties, graphene has been extensively studied in different research fields, such as in nanoelectronic devices, sensors, conductors, solar cells, etc. [18]. GO is investigated in biological and biomedical fields due to its biocompatibility along with its excellent physical properties [19,20,21,22] for drug delivery [22,23,24,25], such as in the delivery of oridonin and methotrexate [26], the delivery of paclitaxel [27], and the delivery of SiRNA [28], etc.

Polyethylene glycol (PEG) possesses a low toxicity and higher solubility in aqueous solutions and has biocompatibility [29,30] and elimination through renal and hepatic routes [30]. The FDA has approved PEG for human use and currently various pharmaceutical formulations of PEG are available. Therefore, PEGylated GO (PEG-GO) is expected to be soluble in biological systems as well as being a safe and stable carrier for the delivery of different types of drugs [31].

Drugs with different molecular structures behave differently during processing. Aromatic ring-containing drugs are most often water insoluble [32], which causes a decrease in the bioavailability of the drugs and worsens the subsequent therapeutic outcomes. Previously, it was reported that the drug loading on GO occurs via π–π interaction [13,33,34,35], hence the number of π bonds (e.g., aromatic ring) in the drug molecule may enhance the drug loading on GO. Therefore, GO can be a promising carrier for the delivery of an aromatic ring containing insoluble drugs.

In this study, drugs with different molecular structures and properties were investigated during processing with a graphene oxide-based nano-drug delivery system. The impact of the process parameters on the average size of the drug-loaded NGO-PEG particles was investigated using design of experiment (DoE). PEG 6000 was used to functionalize or coat the nano graphene oxide particles through physical interactions instead of chemical processing. The solid-state characterization of drug-loaded NGO-PEG suspensions was performed using scanning electron microscopy (SEM), Fourier transform infrared (FTIR) spectroscopy, differential scanning calorimetry (DSC), and X-ray diffraction (XRD). Furthermore, the percentage of drug loading according to the molecular structure of drugs (aromatic ring number) on the NGO-PEG nanoparticles was also investigated.

## 2. Results and Discussion

### 2.1. Particle Size Distribution Analysis and PDI

It is crucial to measure and control the droplet size of the nanosuspensions to maintain quality and performance. The droplet size could influence the rate and extent of the drug release from the nanosuspensions as well as improve the stability. A higher polydispersity (PDI) value refers to the lower uniformity of the nanosuspension droplet size. The AMP-loaded NGO-PEG suspensions prepared in this study were in the range of 200 nm to 1.3 µm, which was characterized by the DLS method (Table 1). The largest droplet sizes were observed in batch number four (1356 nm). with the highest PDI value of 0.88. The droplet sizes considerably dropped down to 209.10 nm, with the lowest PDI value of 0.18 (monodispersed). However, precipitation was observed for the acetaminophen-loaded GO-based nanosuspension batches prepared using the longer stirring time (60 min), therefore the average particle size of these batches was not determined and included in Table 1. It should be noted that the performance of the nanosuspensions is governed by their fate in the gastrointestinal (GI) tract, rather than the particle size of the initial dispersion.

### 2.2. Effect of Process Parameters on the Average Size of GO Nanoparticles

**Figure 1:**
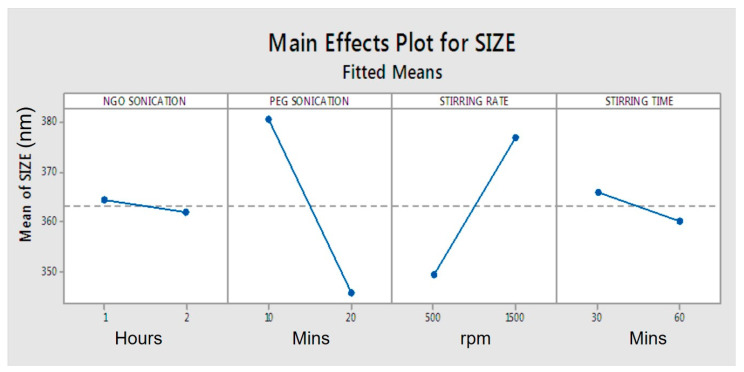
Main effect plots for the impact of process parameters on average particle size.

**Figure 2:**
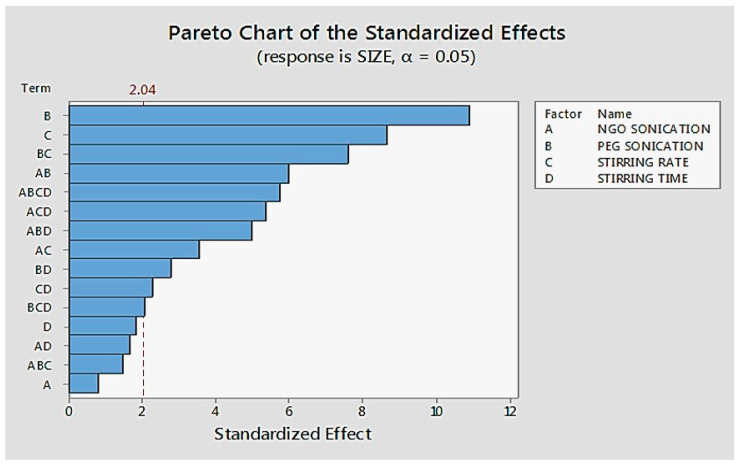
Pareto plot showing the impact of process parameters on the average particle size of the AMP loaded NGO-PEG suspension.

The process parameters’ PEGylation sonication times and stirring rates were found to have a major impact on the average size of the drug-loaded NGO-PEG particles (Figure 1 and Figure 2). The average particle sizes for the drug-loaded NGO-PEG particles were the lowest (AMP: 240 ± 5 nm, DIC: 306 ± 10 nm, MTX: 825 ± 60 nm) when processed at a high level of processing conditions (except stirring time). However, acetaminophen (AMP)-loaded NGO-PEG nanoparticles showed the lowest average size compared to the diclofenac (DIC)- and methotrexate (MTX)-loaded nanoparticles using similar processing conditions. The results suggest that drugs with different molecular structures processed using similar conditions behave differently. This phenomenon is likely related to the brittleness characteristics of acetaminophen compared to other drugs [36]. This phenomenon also may be linked to the different levels of supersaturations generated during the processing of different drugs, leading to the differences in the nucleation rate and the particle size distribution. These results suggest that drugs with different molecular structures and properties will behave differently during processing. The longer duration of sonication exhibited a lower average particle size for nanosuspensions (AMP: 240 ± 5 nm, DIC: 306 ± 10 nm) compared to the shorter duration of sonication (AMP: 290 ± 12 nm, DIC: 362 ± 6 nm). Stirring at a high rate resulted in a lower average particle size for the nanosuspensions (AMP: 240 ± 5 nm, DIC: 306 ± 10 nm) than stirring at a low rate (AMP: 273 ± 11 nm, DIC: 370 ± 15 nm).

### 2.3. Drug Loading Efficiency

**Figure 3:**
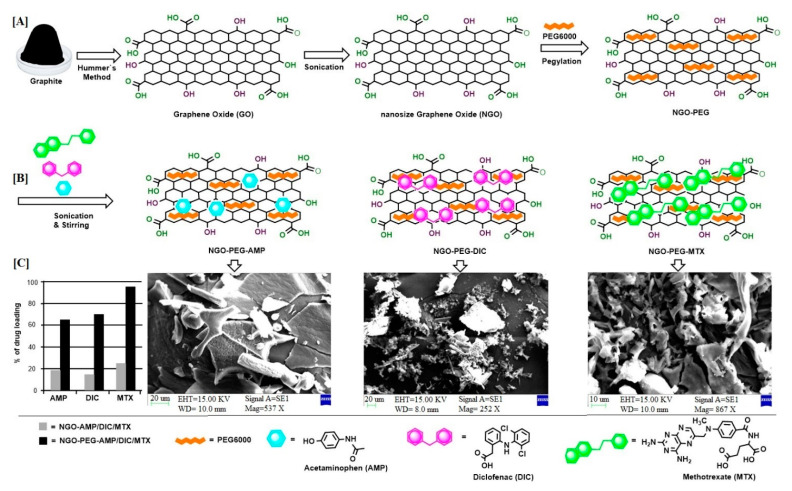
Schematic illustration showing the impact of the molecular structure of the drugs on the drug loading efficiency of NGO-PEG particles: (**A**) preparation of NGO-PEG, (**B**) loading of drugs onto NGO-PEG with different numbers of aromatic rings, (**C**) bar diagram showing the percentage of drug loading and SEM micrographs of drug-loaded NGO-PEG particles.

The research suggests that drug loading on GO occurs via π-π interaction [13], and therefore the number of π bonds (e.g., aromatic ring) in drug molecules may facilitate drug loading on graphene oxide. As such, three different drugs including AMP, DIC and MTX, with 1, 2, and 3 aromatic rings, respectively, were loaded onto NGO-PEG particles. The results shown in Figure 3 suggest that the drug molecule with a greater number of aromatic rings shows higher drug-loading (MTX: 95.6 ± 4.3%; 14.3 mg) compared to the drug with fewer aromatic rings (DIC: 70.5 ± 1.7%; 10.6 mg, AMP: 65.5 ± 1.5%; 9.8 mg) (Figure 3) [13]. This result suggests that drugs with different molecular structures processed using similar conditions behave differently during loading on the NGO-PEG particles. However. drugs loaded onto non-PEGylated NGO particles showed very low percentages of loading (Figure 3).

### 2.4. SEM

**Figure 4:**
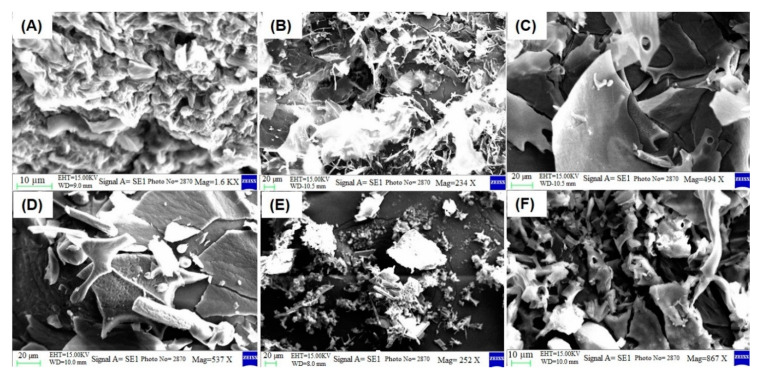
SEM micrographs of (**A**) GO, (**B**) NGO, (**C**) NGO-PEG, (**D**) AMP-loaded NGO-PEG, (**E**) diclofenac (DIC)-loaded NGO-PEG, and (**F**) methotrexate (MTX)-loaded NGO-PEG.

The SEM images (Figure 4A–C) show the morphology of GO, NGO, and PEGylated NGO prepared from GO in this study. The NGO was likely functionalized or coated by the PEG 6000 through physical interaction, which was evident from the smooth surface of the PEG-NGO (Figure 4C). The SEM data (Figure 4D–F) also show that three different drugs were loaded onto the NGO-PEG and the highest drug loading was observed for MTX (Figure 4F).

### 2.5. FTIR

**Figure 5:**
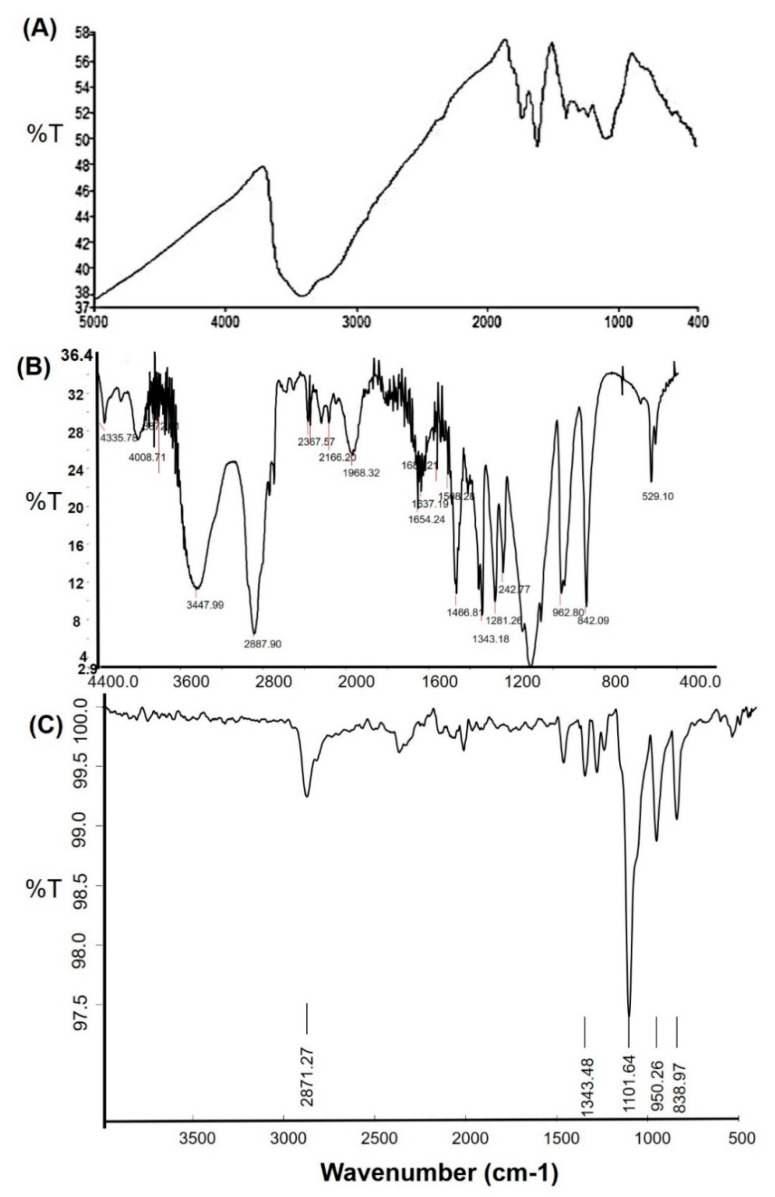
Fourier transform infrared (FTIR) spectra of (**A**) GO, (**B**) PEG 6000 and (**C**) PEGylated NGO.

The FTIR spectra of NGO, PEG 6000, and PEGylated NGO are displayed in Figure 5. The FTIR spectrum of NGO (Figure 5A) showed characteristic peaks at ⁓3400 cm^−1^ and ⁓1720 cm^−1^ for –OH and the C=O functional group, respectively [37,38]. The FTIR spectrum of the PEG 6000 (Figure 5B) also showed strong intense bands at the wavenumbers of ⁓1102 cm^−1^ (C–O–C stretch), ⁓2888 cm^−1^ (C–H stretch), and ⁓3448 cm^−1^ (O–H stretch) [39].

Two of these major intense peaks in Figure 5B were also present in Figure 5C for PEGylated NGO at a ⁓1102 cm^−1^ (C–O–C stretch), and ⁓2888 cm^−1^ (C–H stretch) wavenumber. Therefore, the FTIR data (Figure 5) suggest that the NGO was likely functionalized or coated by PEG 6000 using the sonication method, which was also evident from the SEM micrographs (Figure 4C). GO and PEG 6000 contain many carboxylate and hydroxyl groups. Therefore, these groups were good candidates for forming hydrogen bonds. In addition, PEG 6000 is a commonly used stabilizer which also may take part in van der Waals interactions among GO, drugs and solvents. Therefore, PEG 6000 may have contributed to the activation of GO physically through these interactions.

### 2.6. DSC

**Figure 6:**
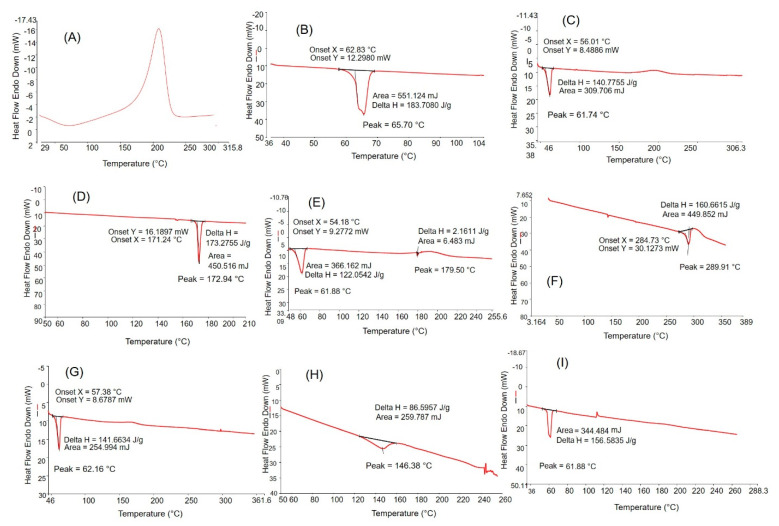
DSC thermograms of (**A**) NGO, (**B**) PEG 6000, (**C**) NGO-PEG, (**D**) AMP, (**E**) AMP loaded NGO-PEG, (**F**) DIC, (**G**) DIC-loaded NGO-PEG, (**H**) MTX, and (**I**) MTX-loaded NGO-PEG.

The DSC result illustrated in Figure 6A showed a sharp exothermic peak at ⁓200 °C which was possibly attributed to the reduction of GO (NGO). An exothermic peak for GO at ⁓196 °C was also observed by Traina and Pegoretti [40]. PEG 6000 (Figure 6B) showed an endothermic melting transition at 66.7 °C with ΔH = 183.70 J/g. The DSC thermogram of NGO-PEG (Figure 6C) showed a sharp endothermic peak at 61.74 °C and a diffuse broad exothermic peak in the range of 170–220 °C which is probably related to the endothermic melting of PEG 6000 and reduction of GO, respectively [40]. These results suggest that possibly NGO was functionalized by PEG 6000.

The DSC thermograms showed strong endothermic melting peaks for all three drugs used in this study (Figure 6D,F,H). The drug-loaded NGO-PEG did not show any endothermic melting peaks for the drugs, except an endothermic melting peak for PEG 6000 at a slightly lower temperature (61.8–62.16 °C) compared to the pure PEG 6000. The absence of drug endothermic peaks may indicate that the drugs are dispersed into the NGO-PEG particles mostly or the drugs are likely converted into the amorphous state during processing and loading on the NGO-PEG particles. This phenomenon was also observed in previous studies when PEG 6000 was used in developing solid dispersions of different drugs [41,42,43,44].

### 2.7. X-ray Diffraction (XRD)

**Figure 7:**
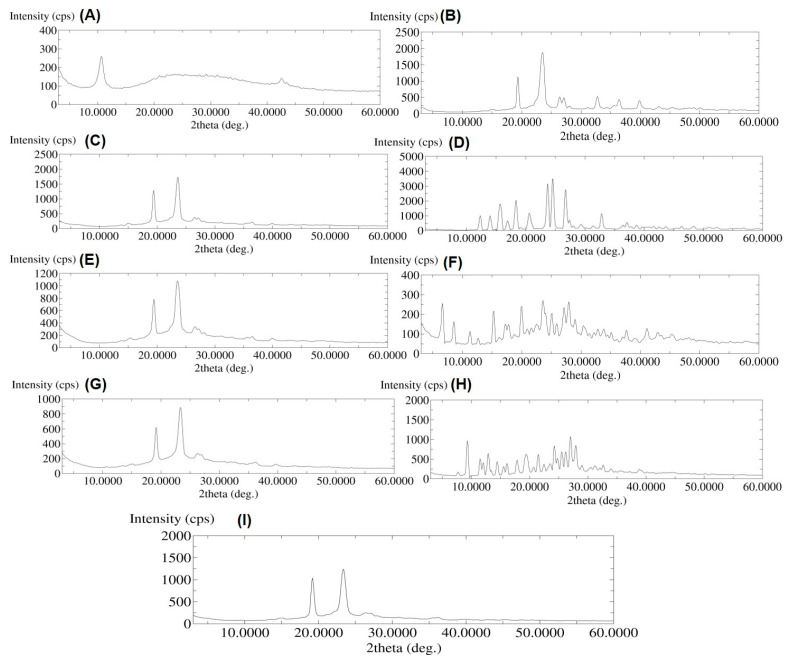
PXRD pattern of (**A**) NGO, (**B**) PEG 6000, (**C**) NGO-PEG, (**D**) AMP, (**E**) AMP loaded NGO-PEG, (**F**) DIC, (**G**) DIC-loaded NGO-PEG, (**H**) MTX, and (**I**) MTX-loaded NGO-PEG.

The X-ray diffraction (XRD) data (Figure 7A) showed a sharp intense peak at a 2Ɵ value of ⁓10° for NGO, which was also observed by other researchers for GO [45,46] with a broad peak in the range of 20–30° 2θ, indicating the amorphous nature of the GO while converting into NGO by sonication. Figure 7B shows a crystalline XRD pattern with a sharp, intense peak at around 19.40° and 23.34° 2θ for PEG 6000. This result also coincides with the XRD pattern observed by Valizadeh et al. for PEG 6000 [46]. The XRD pattern for the functionalized NGO-PEG showed only the major peak of PEG 6000; however, no sharp peak was observed at 2θ value of ⁓10°. The DSC result showed similar characteristics for NGO-PEG which indicates that the functionalization or coating of NGO likely has occurred by PEG 6000. Since 5 mg of PEG 6000 has been used per mL of the NGO suspension, it is assumed that the PEG 600 might have coated the NGO particles predominantly.

This result also suggests that NGO probably was dispersed in the PEG 6000 and exhibited only the major peaks of PEG 6000. Pure AMP, DIC and MTX showed sharp crystalline XRD patterns (Figure 7D,F,H); however, the drug loaded NGO-PEG showed a sharp, intense peak at 19.40° and 23.34° 2θ only, which are the major peaks for PEG 6000. These results suggest that drugs loaded onto NGO-PEG may be dispersed into PEG 6000 or become amorphous during the preparation process. Therefore, no sharp major peaks for all of the three drugs were observed from the XRD pattern of the drug-loaded NGO-PEG particles.

## 3. Materials and Methods

### 3.1. Materials

Graphite was purchased from Qualikems Fine Chem Pvt. Ltd. (Gujarat, India) with an average particle diameter of 4 micrometers. Sulfuric acid (98%), ortho phosphoric acid (85%), and hydrochloric acid (37%) were purchased from Merck (Darmstadt, Germany). Potassium permanganate and hydrogen peroxide (30%) were purchased from Sigma, (Darmstadt, Germany). Acetaminophen, Diclofenac and PEG 6000 were obtained from the Department of Pharmaceutical Sciences Lab, North South University, (Dhaka, Bangladesh). Methotrexate was provided by Popular Pharmaceuticals Limited, (Dhaka, Bangladesh), as a gift.

### 3.2. Methods

#### 3.2.1. Preparation of Graphene Oxide (GO)

Graphene oxide (GO) was prepared by a modified Hummer method [47]. Three grams of graphite and 18 g of potassium permanganate (1:6 by weight) were thoroughly mixed together in a 1 L beaker. Subsequently, 360 mL of 98% sulphuric acid was poured into a 500 mL beaker and then 40 mL of 75% phosphoric acid (9:1 ratio) was added. The acids were added to the graphite and potassium permanganate mixture while stirring (approximately 50 rpm) with a glass rod (slowly and in a clockwise motion) at room temperature (25 °C). The mixture was then placed on a hot plate and stirred at 50 °C for 12 h. The mixture was allowed to cool at room temperature. The ice, prepared using deionized water, was transferred into a large glass container. The graphite acid mixture was then poured over the ice in the large glass container. Three milliliters of 30% hydrogen peroxide was added to the mixture and stirred to obtain GO. Two liters of tap water was added to the GO mixture and left for two days to settle down. The excess water was then removed and the process was repeated twice. The precipitate was collected and kept in an open beaker so that the remaining water could evaporate at room temperature. The precipitate was then rinsed using 10% HCl and deionized water, respectively, and dried in ambient conditions.

#### 3.2.2. Preparation of NGO Particles

NGO batches of 50 mL each with a concentration of 1 mg/mL were prepared by the sonication method. The amount of GO required for each 50 mL suspension was calculated from the desired concentrations. Then, the GO was added to 50 mL of distilled water. This GO suspension was then sonicated using a Cole-Parmer 130-Watt ultrasonic processor (248 mm × 89 mm × 318 mm) at 80% amplification. The process was run in ambient conditions (25 °C temperature) for two hours.

#### 3.2.3. Preparation of PEGylated NGO

The NGO suspension was sonicated using a Cole-Parmer 130-Watt ultrasonic processor (248 mm× 89 mm × 318 mm) with 250 mg of PEG 6000. The process was run in an ambient condition (25 °C) for two hours. Then the suspension was placed into a water bath at 85 °C for 4 h. Samples were collected from the different batches and analyzed using the dynamic light scattering (DLS) method.

#### 3.2.4. Preparation Process of Drug Loaded PEGylated NGO Using Design of Experiment (DoE)

The 15 mL suspensions of drugs (acetaminophen, diclofenac, and methotrexate) at a concentration of 1 mg/mL were prepared using deionized water and then added to 15 mL of the PEGylated NGO suspension. Next, the whole suspension was stirred using a magnetic stirrer at different stirring rates and for different stirring time periods. The effect of the process parameters (PPs) on the average particle size of the acetaminophen-loaded NGO-PEG particles was evaluated using DoE (Table 2). Each of the factors was studied at two levels (high = H and low = L), where preliminary studies were utilized to identify the parameter range for the detailed study. The factors used for the design of experiment (DoE) in this study were selected based on previous research and a few pilot-scale batch production assessments.

A full factorial experimental design (Table 2) implementing four process parameters was used. The four process parameters included: (1) the sonication time to produce nano graphene oxide; (2) the sonication time to prepare PEGylated nano graphene oxide; (3) the rate of stirring at which the drug is incorporated in the PEGylated nano graphene oxide; and (4) the duration of stirring of the drug and PEGylated nano graphene oxide at two levels, high and low, using acetaminophen as a drug (Table 2). The high and low values of the four process parameters are as follows: the duration of sonication to produce NGO (high = 2 h; low = 1 h), the duration of sonication for PEGylated NGO (high = 20 min; low = 10 min), the stirring rate (high = 1500 rpm; low = 500 rpm), and the stirring time (high = 60 min; low = 30 min). The PEGylated nanographene oxide-based delivery of other drugs (diclofenac and methotrexate) was prepared using optimized conditions found after the experimental design study.

#### 3.2.5. Drug Loading Efficiency

Percentages of drug loading for graphene oxide-based nano suspensions were sampled in eppendorf tubes and centrifuged at 9800× *g* for 30 min to separate the unbound drug from the drug-loaded NGO-PEG. Both the precipitate and the supernatant were analyzed using UV spectroscopy to determine the concentrations of the bound and unbound drugs, respectively. The UV tests were performed for acetaminophen at 243 nm, diclofenac at 340 nm, and methotrexate at 303 nm. The calibration curve equation and correlation of coefficient values are: for acetaminophen, y = 0.0693x + 0.0789, r^2^ = 0.996; for diclofenac, y = 0.0074x + 0.125, r^2^ = 0.992; and for methotrexate, y = 0.086x + 0.002, r^2^ = 0.998. Five different concentrations were used for all three of the different drugs to make the calibration curve, which are 5, 10, 15, 20, and 25 µg/mL.

#### 3.2.6. Particle Size Analysis and Determination of Polydispersity Index (PDI) by Dynamic Light Scattering (DLS)

The particle size distribution and PDI of all the representative graphene oxide (GO)-based nano preparations were determined by the dynamic light scattering (DLS) method using the Malvern Zetasizer Nano-ZS (Malvern Instruments, UK) at a 90° scattering angle. The nanosuspensions were diluted at a ratio of 1:100 *v*/*v* (nanosuspensions: deionized water) and mixed for 1 min using a VM1 vortex mixer (Boronia, Australia) before the analysis at 25 °C. The analysis was performed in triplicate and the average value was calculated from the data collected 10 times in the study.

#### 3.2.7. Scanning Electron Microscopy (SEM)

The graphene oxide (GO)-based nano preparations were visualized using a (Carl Zeiss AG, Jena, Germany) scanning electron microscope (SEM). The samples were analyzed at a variety of magnifications and captured in high-resolution images onto a personal computer. The samples were distributed onto double-sided adhesive carbon tapes, which were attached to SEM specimen mounts. The specimens were sputter-coated by a Jeol JFC-1600 auto fine coater for 2 min.

#### 3.2.8. Fourier Transform Infrared (FTIR) Spectroscopy

An FTIR analysis was conducted to examine possible interactions present between GO and GO-based nano preparations. The chemical properties were obtained and the complexation of the powdered lyophilized samples was performed using the FTIR Spectrum BX from Perkin Elmer LLC (Hopkinton, MA, USA). Pure GO and graphene oxide-based nano preparations (solid powders) were compressed for 5 min at 5 bars on a KBr press and the spectra were scanned on the wavenumber range of 400–4000 cm^−1^.

#### 3.2.9. Differential Scanning Calorimetry (DSC)

The thermal characteristics of the powdered samples of NGO, PEG 6000, NGO-PEG, pure drugs, and freeze-dried nanosuspensions of drug-loaded NGO-PEG were characterized by DSC (DSC-60, Shimadzu, Kyoto, Japan). Samples (2–5 mg) were hermetically sealed in aluminum sample pans and heated at a scanning rate of 10 °C/min over a temperature range of 25–360 °C. All the samples were analyzed in triplicate and the temperature scale was calibrated using a pure indium standard (melting point of 156.6 °C).

#### 3.2.10. X-ray Diffraction (XRD)

The X-ray diffraction (XRD) was used to characterize the solid-state form of NGO, PEG 6000, NGO-PEG, pure drugs, and freeze-dried nanosuspensions of drug loaded NGO-PEG using a Rigaku multiflex diffractometer (Rigaku Corporation, Tokyo, Japan). The X-ray source was Ni filtered CuK-alpha radiation (wavelength 1.5418 A). The X-ray tube was run at a power of 40 kV, 40 mA. The analyses were performed over a 2θ range of 3–60° with an angular increment of 0.50 °/min and a scan step time of 1.0 s.

## 4. Conclusions

The results of this study suggest that drugs with different molecular structures processed using similar conditions behave differently in terms of average particle size and loading efficiency on NGO-PEG particles. The NGO-PEG suspension processed with AMP showed the lowest average particle size compared to DIC and MTX, which is probably related to the molecular structure and, more specifically, the different properties (solubility, mechanical behavior) of different solids (drugs). The DoE study results suggest that PEGylation sonication time and stirring rate are two major parameters which proved to have a marked impact on the average particle size of the drug-loaded NGO-PEG. This result also suggests that PEGylation sonication time should be increased (>20 min) to achieve NGO-PEG particles with an average size of around 100 nm, which indicates the advantage of using DoE during the processing of NGO-PEG particles. The molecular structure of the drugs, particularly the number of aromatic rings present in the structure, affects the drug loading on NGO-PEG. MTX with three aromatic rings exhibited the highest loading on NGO-PEG particles compared to drugs with fewer aromatic rings, which was also evident in the SEM micrographs. The FTIR, DSC, and XRD data suggest that functionalization or the coating of NGO was performed using PEG 6000, which also suggests that the drugs were dispersed into PEG 6000 or became amorphous when loaded onto NGO-PEG particles during processing. PEGylation of NGO by physical interactions offers a simple and less time-consuming process for developing a functionalized NGO-PEG based drug delivery system. This study also suggests that a GO-based nano drug delivery system can be used as a promising carrier for the delivery of an aromatic ring containing insoluble drugs.

## Figures and Tables

**Table 1:** Average particle size of acetaminophen-loaded NGO-PEG suspensions prepared using DoE.

No.	NGO Sonication Time (hrs)	PEGylation Sonication Time (mins)	Stirring Time (mins)	Stirring Rate (rpm)	Average Size (nm)	Standard Deviation (SD)	PDI
1	2	20	30	1500	209.1	3.0	0.18
2	2	20	30	500	1129.0	35.4	0.76
3	2	10	30	1500	880.8	31.2	0.40
4	2	10	30	500	1356.0	56.0	0.83
5	1	20	30	1500	278.3	8.3	0.37
6	1	20	30	500	641.9	14.3	0.26
7	1	10	30	1500	749.9	27.8	0.63
8	1	10	30	500	834.5	21.0	0.80

**Table 2:** Design of experiment (DoE) for the preparation of acetaminophen (AMP)-loaded PEGylated nano graphene oxide (NGO) batches.

Batch	Sonication Duration to Produce Nano GO	Sonication Duration to Produce PEGylated Nano GO	Stirring Rate	Stirring Time
1	H	H	H	H
2	H	H	H	L
3	H	H	L	L
4	H	L	L	L
5	L	L	L	L
6	L	L	L	H
7	L	L	H	H
8	L	H	H	H
9	H	L	H	H
10	H	L	H	L
11	H	L	L	H
12	L	H	L	H
13	L	H	H	L
14	L	L	H	L
15	H	H	L	H
16	L	H	L	L

## Data Availability

Not applicable.

## Sample Availability

Samples of the compounds are not available from the authors.
